# BIX-01294 sensitizes renal cancer Caki cells to TRAIL-induced apoptosis through downregulation of survivin expression and upregulation of DR5 expression

**DOI:** 10.1038/s41420-018-0035-8

**Published:** 2018-02-20

**Authors:** Seon Min Woo, Seung Un Seo, Kyoung-Jin Min, Taeg Kyu Kwon

**Affiliations:** Department of Immunology, School of Medicine, Keimyung University, 1095 Dalgubeoldaero, Dalseo-Gu, Daegu 42601 South Korea

## Abstract

BIX-01294 (BIX), a G9a histone methyltransferase inhibitor, has been reported for its anti-proliferative and anticancer activities against various cancer cell lines. In this study, we investigated whether BIX could sensitize TRAIL-mediated apoptosis in various cancer cells. Combined treatment with BIX and TRAIL markedly induced apoptosis in human renal carcinoma (Caki, ACHN, and A498), breast carcinoma (MCF-7), and lung carcinoma (A549) cells. In contrast, BIX and TRAIL co-treatment did not induce apoptosis in normal cells, specifically mouse kidney cell (TCMK-1) and human skin fibroblast (HSF). BIX downregulated protein expression levels of XIAP and survivin at the post-translational level. Overexpression of survivin markedly blocked combined BIX and TRAIL treatment-induced apoptosis, but XIAP had no effect. Furthermore, BIX induced upregulation of DR5 expression at the transcriptional levels, and knockdown of DR5 expression using small interfering RNAs (siRNAs) markedly attenuated BIX and TRAIL-induced apoptosis. Interestingly, siRNA-mediated G9a histone methyltransferase knockdown also enhanced TRAIL-induced apoptosis in Caki cells. However, knockdown of G9a did not change expression levels of XIAP, survivin, and DR5. Therefore, BIX-mediated TRAIL sensitization was independent of histone methyltransferase G9a activity. Taken together, these results suggest that BIX facilitates TRAIL-mediated apoptosis via downregulation of survivin and upregulation of DR5 expression in renal carcinoma Caki cells.

▶ BIX facilitates TRAIL-mediated apoptosis in human renal carcinoma Caki cells.

▶ Downregulation of survivin contributes to BIX plus TRAIL-induced apoptosis.

▶ Upregulation of DR5 is involved in BIX plus TRAIL-mediated apoptosis.

▶ BIX-mediated TRAIL sensitization is independent of ROS production.

## Introduction

Histone methylation is one of the major epigenetic modifications and plays an important role in biological processes by regulating transcriptional activity of a target gene^1–3^. Histone methyltransferase G9a, also known as euchromatic histone-lysine *N*-methyltransferase 2 (EHMT2), is a key methyltransferase for histone H3 lysine 9 (H3K9)^4^. Moreover, it is overexpressed in malignant cells, including breast cancer^5^, prostate cancer^6^, bladder cancer^7^, and colorectal cancer^8^, and elevated levels of G9a are associated with cell survival, proliferation, and metastasis^9–11^. BIX-01294 (BIX) is a potent G9a inhibitor that induces demethylation of H3K9^12^. In a previous study, BIX inhibited cell proliferation and induced mitochondrial apoptosis through downregulation of Bcl-2 expression in lung adenocarcinoma^13^. Additionally, BIX decreased Mcl-1 expression via downregulation of the deubiquitinase USP9X, leading to caspase-dependent apoptosis in bladder cancer cells^14^. In addition, BIX induced autophagy-mediated cell death through production of reactive oxygen species (ROS) in breast cancer^15, 16^ and upregulation of p53 expression in colon cancer^17^. Moreover, BIX inhibits HIF-1α stability and VEGF-mediated angiogenesis in hepatocellular carcinoma^18^.

Tumor necrosis factor (TNF)-related apoptosis-inducing ligand (TRAIL) is a member of the TNF superfamily that selectively triggers apoptosis in cancer cells, but not in normal cells^19^. TRAIL binds death receptor (DR) and forms death-inducing signal complex by recruiting FAS-associated protein death domain and caspase-8, eventually inducing apoptosis^20^. However, many cancer cells present resistance to TRAIL, which is involved in downregulation of DR expression and upregulation of anti-apoptotic proteins, including the Bcl-2 family and inhibitor of apoptosis proteins (IAPs)^21–23^. Therefore, identification of an effective sensitizer is needed.

In this study, we investigated the effect of BIX on TRAIL-mediated apoptosis in human renal carcinoma Caki cells. We demonstrated that BIX sensitizes TRAIL-induced apoptosis through downregulation of survivin expression and upregulation of DR5 expression. These results provide the evidence that BIX could be a novel sensitizer of TRAIL-resistant cancer cells.

## Results

### BIX sensitizes TRAIL-mediated apoptosis in human renal carcinoma Caki cells

**Fig. 1:**
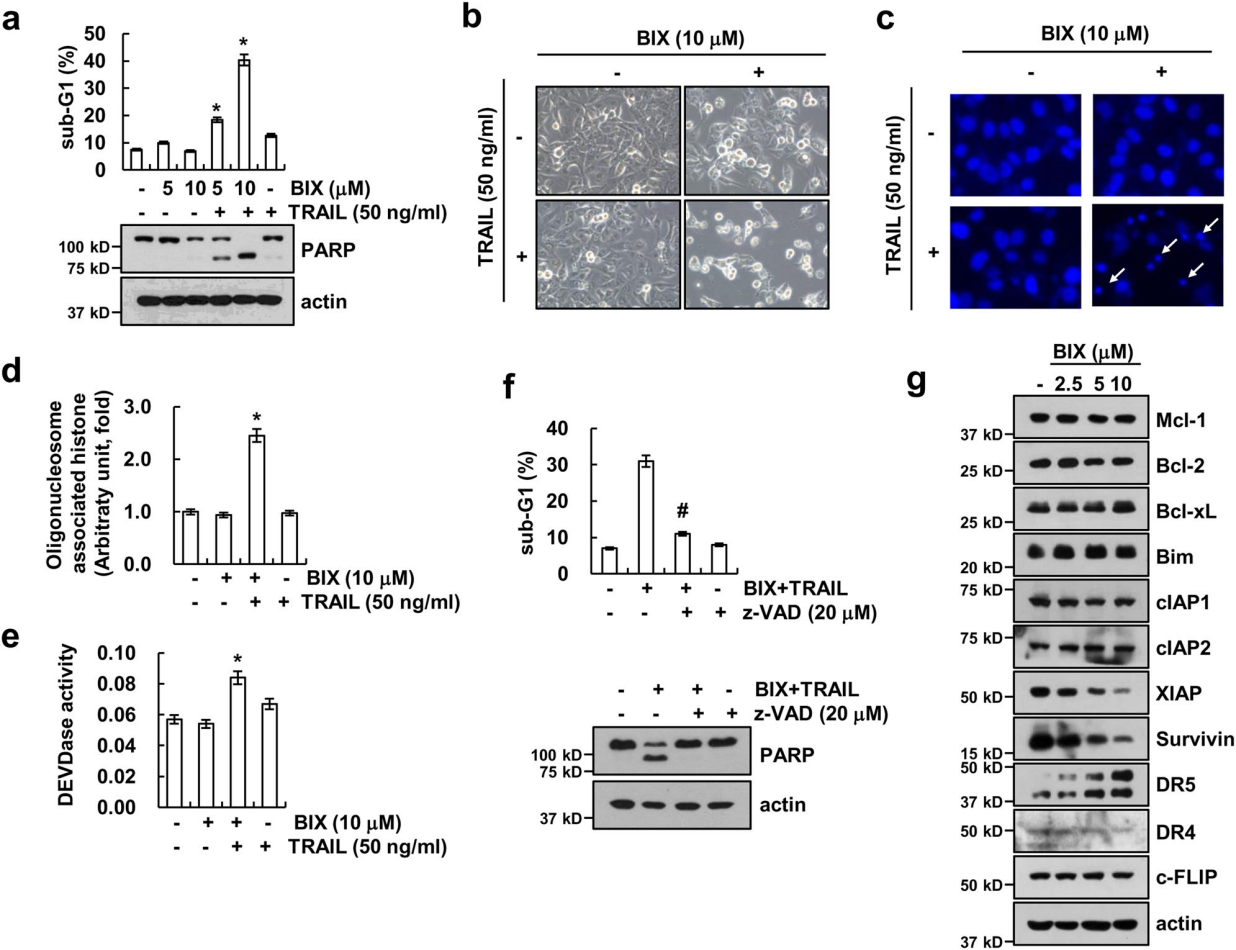
BIX-01294 sensitizes Caki cells to TRAIL-induced apoptosis in caspase-dependent manner. **a** Caki cells were treated with 50 ng/ml TRAIL in the presence or absence of the indicated concentrations of BIX for 24 h. Apoptosis was analyzed as a sub-G1 population by flow cytometry. The protein levels of PARP and actin were determined by western blotting. The level of actin was used as a loading control. **b**–**e** Caki cells were treated with 50 ng/ml TRAIL in the presence or absence of 10 μM BIX for 24 h. The cell morphology was examined using interference light microscopy **b**. The condensation and fragmentation of the nuclei were detected by 4′,6′-diamidino-2-phenylindole staining **c**. The cytoplasmic histone-associated DNA fragments were determined by a DNA fragmentation detection kit **d**. Caspase activities were determined with colorimetric assays using caspase-3 (DEVDase) assay kits **e**. **f** Caki cells were treated with 10 μM BIX plus 50 ng/ml TRAIL for 24 h in the presence or absence of 20 μM z-VAD-fmk (z-VAD). Apoptosis was analyzed as a sub-G1 population by flow cytometry. The protein levels of PARP and actin were determined by western blotting. The level of actin was used as a loading control. **g** Caki cells were treated with the indicated concentrations of BIX for 24 h. The protein levels of Mcl-1, Bcl-2, Bcl-xL, Bim, cIAP1, cIAP2, XIAP, survivin, DR5, DR4, c-FLIP, and actin were determined by western blotting. The level of actin was used as a loading control. The values in **a**, **d**, **e**, and **f** represent the mean ± SD from three independent samples; *p < 0.05 compared with the control. ^#^*p < *0.01 compared with the combined treatment with BIX and TRAIL

As BIX has anticancer effects^13–16^, we investigated whether BIX could enhance TRAIL-induced apoptosis in human renal carcinoma Caki cells. As shown in Fig. 1a, combined treatment with BIX and TRAIL markedly increased the sub-G1 population and cleavage of poly (ADP-ribose) polymerase (PARP). Moreover, combined treatment with BIX plus TRAIL showed typical apoptotic morphologies, chromatin condensation, and DNA fragmentation (Figs. 1b–d). Next, we examined whether activation of caspases plays a major a role in BIX plus TRAIL-induced apoptosis. Combined treatment with BIX and TRAIL-induced caspase-3 activity (Fig. 1e), and treatment with z-VAD-fmk, the pan-caspase inhibitor, completely inhibited BIX plus TRAIL-induced apoptosis and PARP cleavage (Fig. 1f). To identify the underlying mechanisms of TRAIL sensitization by BIX, we checked the expression levels of apoptosis-related proteins. BIX induced downregulation of XIAP and survivin expression, as well as upregulation of DR5 expression, with the expression levels of other proteins remaining unchanged (Fig. 1g). These results indicate that BIX enhances TRAIL-induced caspase-dependent apoptosis in Caki cells.

### Downregulation of XIAP by BIX treatment

**Fig. 2:**
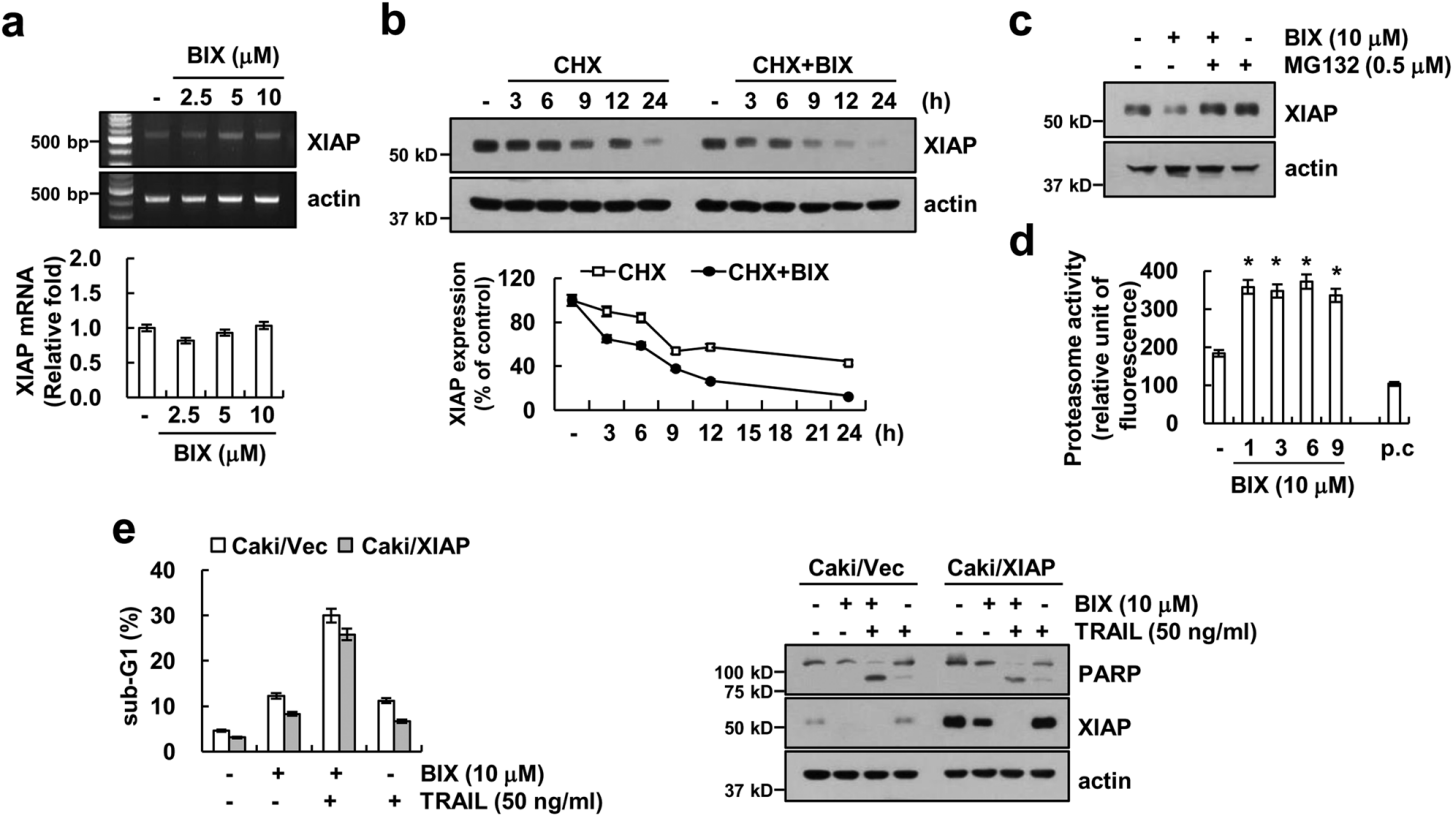
BIX-01294 induces downregulation of XIAP expression at the post-translational level. **a** Caki cells were treated with the indicated concentrations of BIX for 24 h. The mRNA levels of XIAP and actin were determined by RT-PCR (upper panel) and quantitative PCR (qPCR, lower panel). The level of actin was used as a loading control. **b** Caki cells were treated with or without 10 μM BIX in the presence of 20 μg/ml cyclohexamide (CHX) for the indicated time periods. The protein levels of XIAP and actin were determined by western blotting. The level of actin was used as a loading control (upper panel). The band intensity of the XIAP protein was measured using ImageJ (public domain JAVA image-processing program; http://rsb.info.nih.gov/ij, lower panel). **c** Caki cells were pretreated with 0.5 μM MG132 for 30 min and then treated with 10 μM BIX for 24 h. The protein levels of XIAP and actin were determined by western blotting. The level of actin was used as a loading control. **d** Caki cells were treated with 10 μM BIX or MG132 (as a positive control) for the indicated time periods or 6 h (MG132). The cells were lysed, and the proteasome activity was measured, as described in the Materials and methods section. **e** Caki cells were transiently transfected with pEBB/XIAP or vector plasmid. Twenty-four hours after transfection, cells were treated with 50 ng/ml TRAIL in the presence or absence of 10 μM BIX for 24 h. Apoptosis was analyzed as a sub-G1 population by flow cytometry. The protein levels of PARP, XIAP, and actin were determined by western blotting. The level of actin was used as a loading control (lower panel). The values in **a**, **d**, and **e** represent the mean ± SD from three independent samples; *p < 0.05 compared with the control

Next, we investigated how BIX regulates expression of XIAP. BIX induced downregulation of XIAP protein expression (Fig. 1g), but mRNA levels of XIAP were not changed in BIX-treated cells (Fig. 2a). Next, we examined whether BIX could modulate protein stability of XIAP. Caki cells were treated with cycloheximide (CHX), an inhibitor of *de novo* protein synthesis, in the presence or absence of BIX for up to 24 h. BIX plus CHX markedly reduced XIAP expression, compared with CHX alone (Fig. 2b). Therefore, these data indicated that BIX downregulated XIAP protein stability. As ubiquitin–proteasome system is involved in the degradation of proteins^24^, we checked whether the proteasome degradation pathway is associated with BIX-mediated downregulation of XIAP expression. As shown in Fig. 2c, proteasome inhibitor (MG132) markedly reversed BIX-induced downregulation of XIAP protein. Next, to confirm the importance of proteasome activation by BIX, we examined the effect of BIX on activity of the chymotrypsin-like activity of proteasome^25^. As shown in Fig. 2d, BIX increased proteasome activity within 1 h and sustained activity up to 9 h. To examine the role of XIAP downregulation in BIX plus TRAIL-induced apoptosis, we used XIAP-overexpressing Caki cells. Unexpectedly, overexpression of XIAP did not inhibit combined treatment-induced sub-G1 population expansion and PARP cleavage (Fig. 2e).

### Downregulation of survivin by BIX contributes to the sensitization of TRAIL-induced apoptosis

**Fig. 3:**
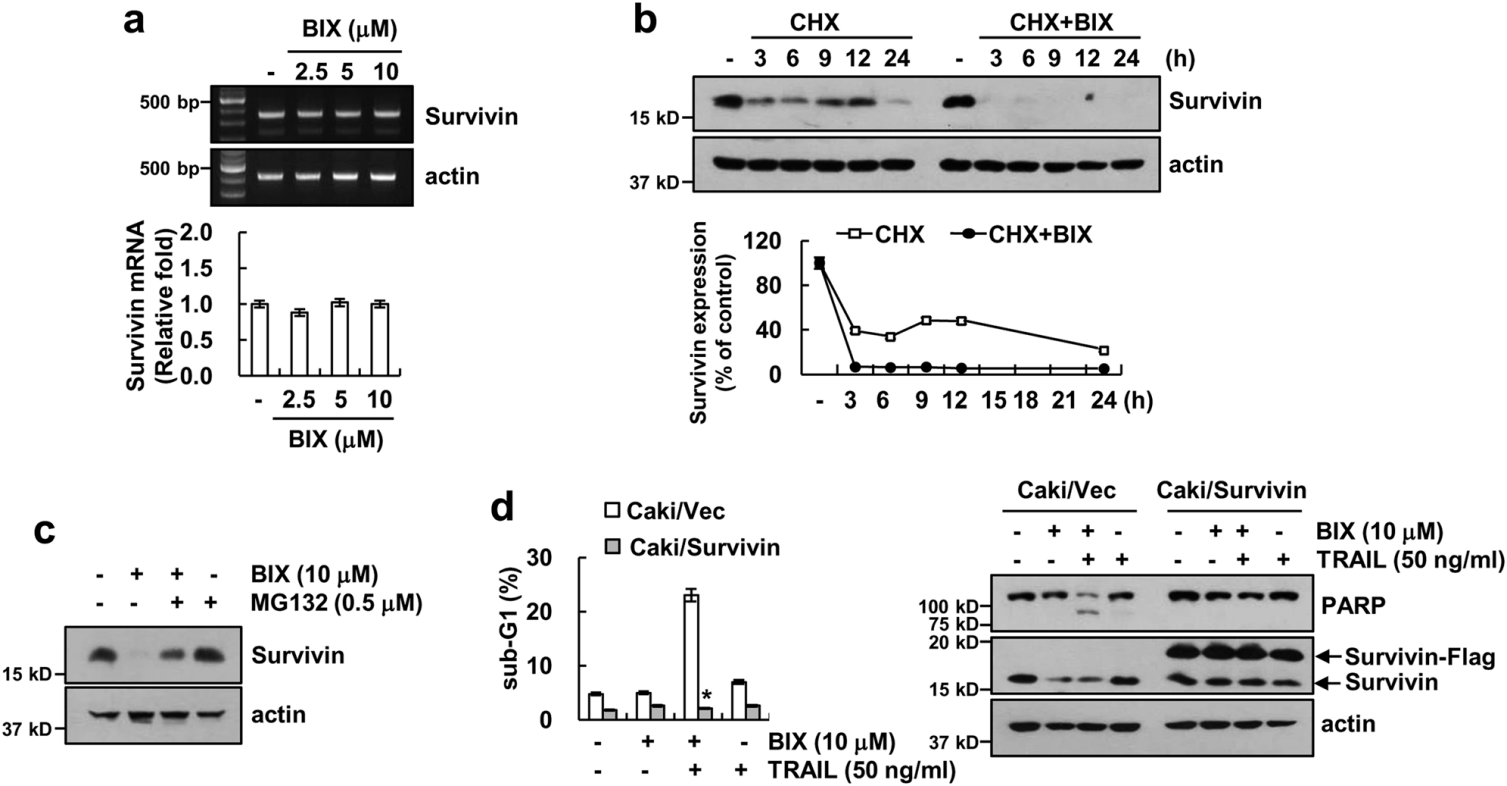
Downregulation of survivin is involved in the induction of BIX-01294 plus TRAIL-induced apoptosis. **a** Caki cells were treated with the indicated concentrations of BIX for 24 h. The mRNA levels of survivin and actin were determined by RT-PCR (upper panel) and quantitative PCR (qPCR, lower panel). The level of actin was used as a loading control. **b** Caki cells were treated with or without 10 μM BIX in the presence of 20 μg/ml cyclohexamide (CHX) for the indicated time periods. The protein levels of survivin and actin were determined by western blotting. The level of actin was used as a loading control (upper panel). The band intensity of the survivin protein was measured using ImageJ (public domain JAVA image-processing program; http://rsb.info.nih.gov/ij, lower panel). **c** Caki cells were pretreated with 0.5 μM MG132 for 30 min and then treated with 10 μM BIX for 24 h. The protein levels of survivin and actin were determined by western blotting. The level of actin was used as a loading control. **d** Vector-transfected cells (Caki/Vec) and survivin-overexpressing cells (Caki/Survivin) were treated with 50 ng/ml TRAIL in the presence or absence of 10 μM BIX for 24 h. Apoptosis was analyzed as a sub-G1 population by flow cytometry (upper panel). The protein levels of PARP, survivin, and actin were determined by western blotting. The level of actin was used as a loading control (lower panel). The values in **a** and **d** represent the mean ± SD from three independent samples; *p < 0.01 compared with the BIX plus TRAIL-treated Caki/Vec

As shown in Fig. 1g, BIX induced downregulation of survivin protein levels, but mRNA levels of survivin did not change (Fig. 3a). BIX also significantly reduced survivin protein stability (Fig. 3b), and proteasome inhibitor reversed BIX-mediated survivin expression (Fig. 3c). To evaluate the functional role of survivin on BIX plus TRAIL-induced apoptosis, Caki cells were transiently transfected with survivin. When survivin was ectopically expressed, the induction of apoptosis and PARP cleavage were significantly inhibited in BIX plus TRAIL-treated cells (Fig. 3d). Therefore, these data indicated that downregulation of survivin may contribute to BIX-mediated TRAIL sensitization rather than XIAP.

### Upregulation of DR5 is involved in BIX plus TRAIL-mediated apoptosis

**Fig. 4:**
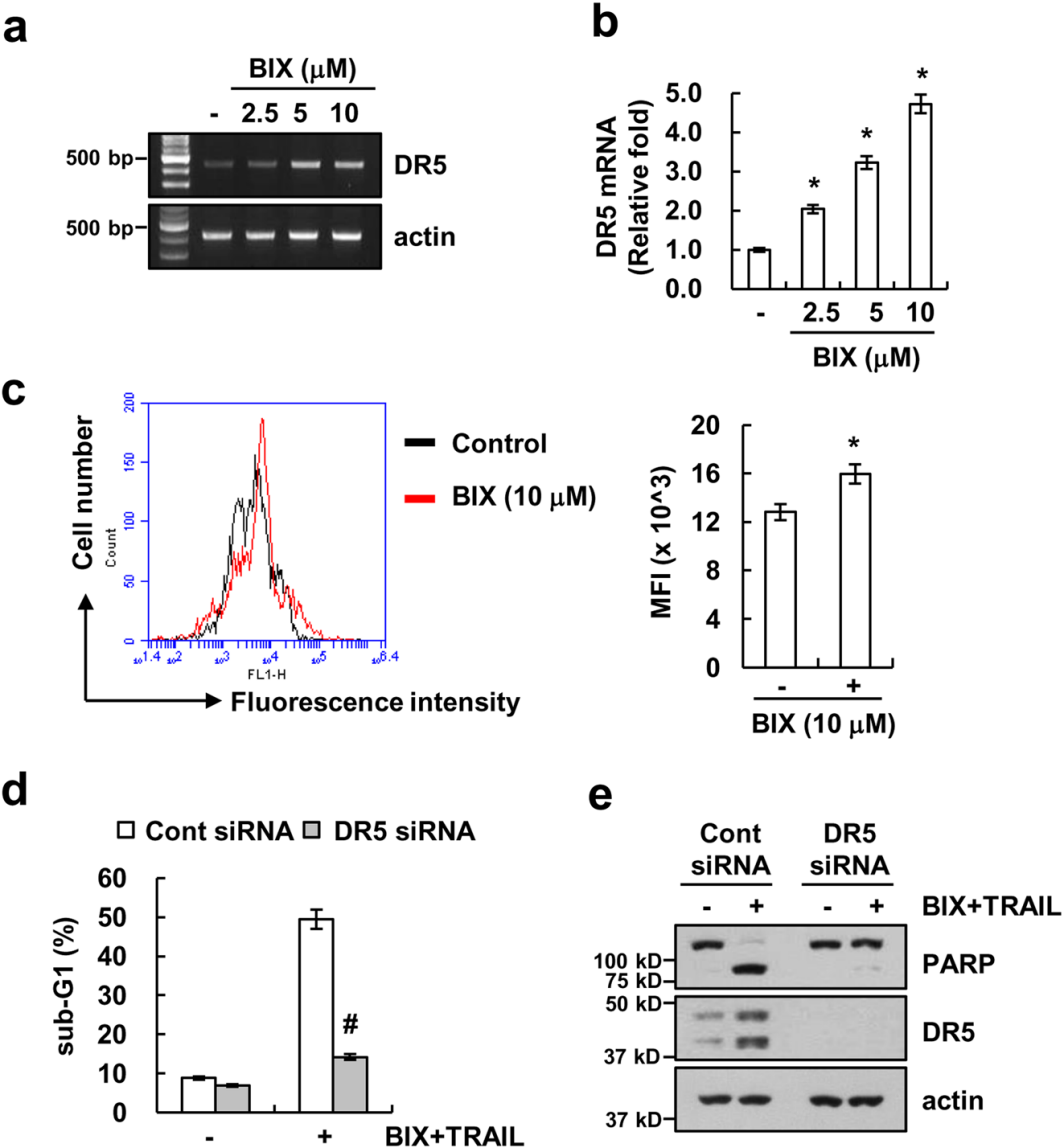
Upregulation of DR5 expression is associated with BIX-01294 plus TRAIL-induced apoptosis. **a**, **b** Caki cells were treated with the indicated concentrations of BIX for 24 h. The mRNA levels of DR5 and actin were determined by RT-PCR **a** and qPCR **b**. **c** Caki cells were treated with 10 μM BIX for 24 h. The cell surface expression level of DR5 was measured by flow cytometry analysis. **d**, **e** Caki cells were transiently transfected control siRNA (Cont siRNA) or DR5 siRNA. Twenty-four hours after transfection, cells were treated with 50 ng/ml TRAIL in the presence or absence of 10 μM BIX for 24 h. Apoptosis was analyzed as a sub-G1 population by flow cytometry **d**. The protein levels of PARP, DR5 and actin were determined by western blotting. The level of actin was used as a loading control **e**. The values in **b**, **c**, and **d** represent the mean ± SD from three independent samples; *p < 0.05 compared with the control. ^#^*p < *0.01 compared with the BIX-01294 plus TRAIL-treated control siRNA

In addition, BIX also increased DR5 protein expression (Fig. 1g). To further explore the underlying mechanism of BIX-induced DR5 expression, we investigated whether BIX induced DR5 mRNA expression. BIX induced upregulation of DR5 mRNA levels and DR5 promoter activity in a dose-dependent manner (Figs. 4a, b). Furthermore, BIX enhanced surface expression levels of DR5 (Fig. 4c). To examine the role of DR5 upregulation in BIX plus TRAIL-mediated apoptosis, Caki cells were transfected with DR5 small interfering RNA (siRNA). Downregulation of DR5 expression by siRNA markedly inhibited apoptosis and PARP cleavage in BIX plus TRAIL-treated cells (Figs. 4d, e). These results suggest that BIX-mediated upregulation of DR5 expression is associated with the induction of TRAIL-induced apoptosis.

### BIX-mediated TRAIL sensitization is independent of ROS production

**Fig. 5:**
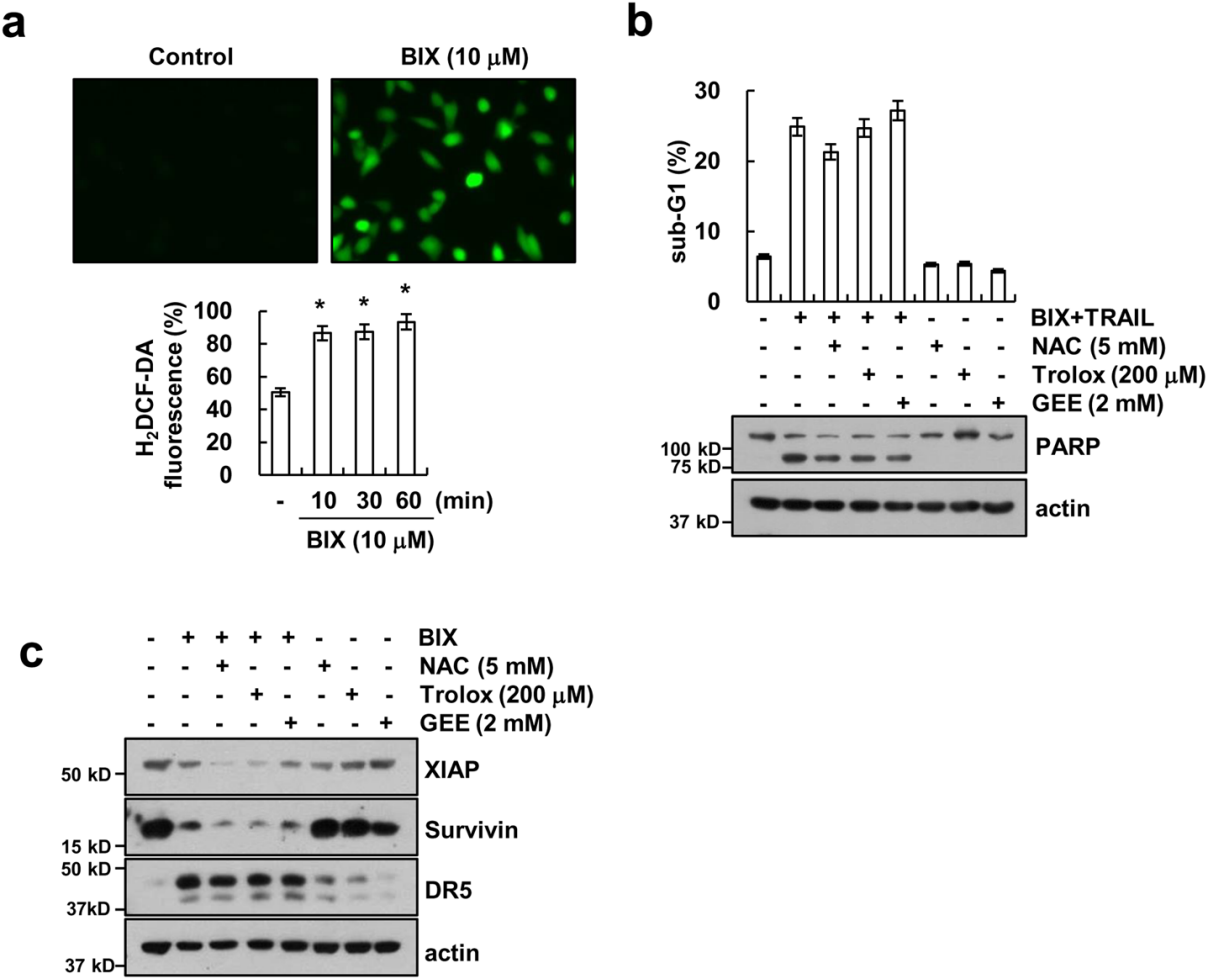
BIX-01294 plus TRAIL-induced apoptosis is independent of ROS signaling in Caki cells. **a** Caki cells were treated with 10 μM BIX for 2 h (upper panel) or the indicated time periods (lower panel) and loaded with a H_2_DCFDA fluorescent dye. The fluorescence intensity was detected by fluorescence microscopy (upper panel) and flow cytometry (lower panel). **b** Caki cells were pretreated with 5 mM NAC, 200 μM trolox, and 2 mM GEE for 30 min, and then treated with 10 μM BIX plus 50 ng/ml TRAIL for 24 h. Apoptosis was analyzed as a sub-G1 population by flow cytometry (upper panel). The protein levels of PARP and actin were determined by western blotting. The level of actin was used as a loading control (lower panel). **c** Caki cells were pretreated with 5 mM NAC, 200 μM trolox and 2 mM GEE for 30 min, and then treated with 10 μM BIX for 24 h. The protein levels of XIAP, survivin, DR5, and actin were determined by western blotting. The level of actin was used as a loading control. The values in **a** and **b** represent the mean ± SD from three independent samples. *p < 0.05 compared with the control

ROS play critical roles in TRAIL sensitization^26, 27^. Therefore, we investigated whether BIX sensitizes TRAIL-mediated apoptosis via ROS production. BIX increased ROS production within 10 min, which was sustained up to 60 min (Fig. 5a). However, ROS scavengers, such as *N*-acetylcysteine (NAC), trolox, and glutathione ethyl ester (GEE), did not affect BIX plus TRAIL-induced apoptosis (Fig. 5b) and modulation of XIAP, survivin, and DR5 expression (Fig. 5c). Thus, these data indicate that BIX-mediated TRAIL sensitization is independent of ROS signaling pathway.

### Inhibition of G9a sensitizes TRAIL-induced apoptosis in Caki cells

**Fig. 6:**
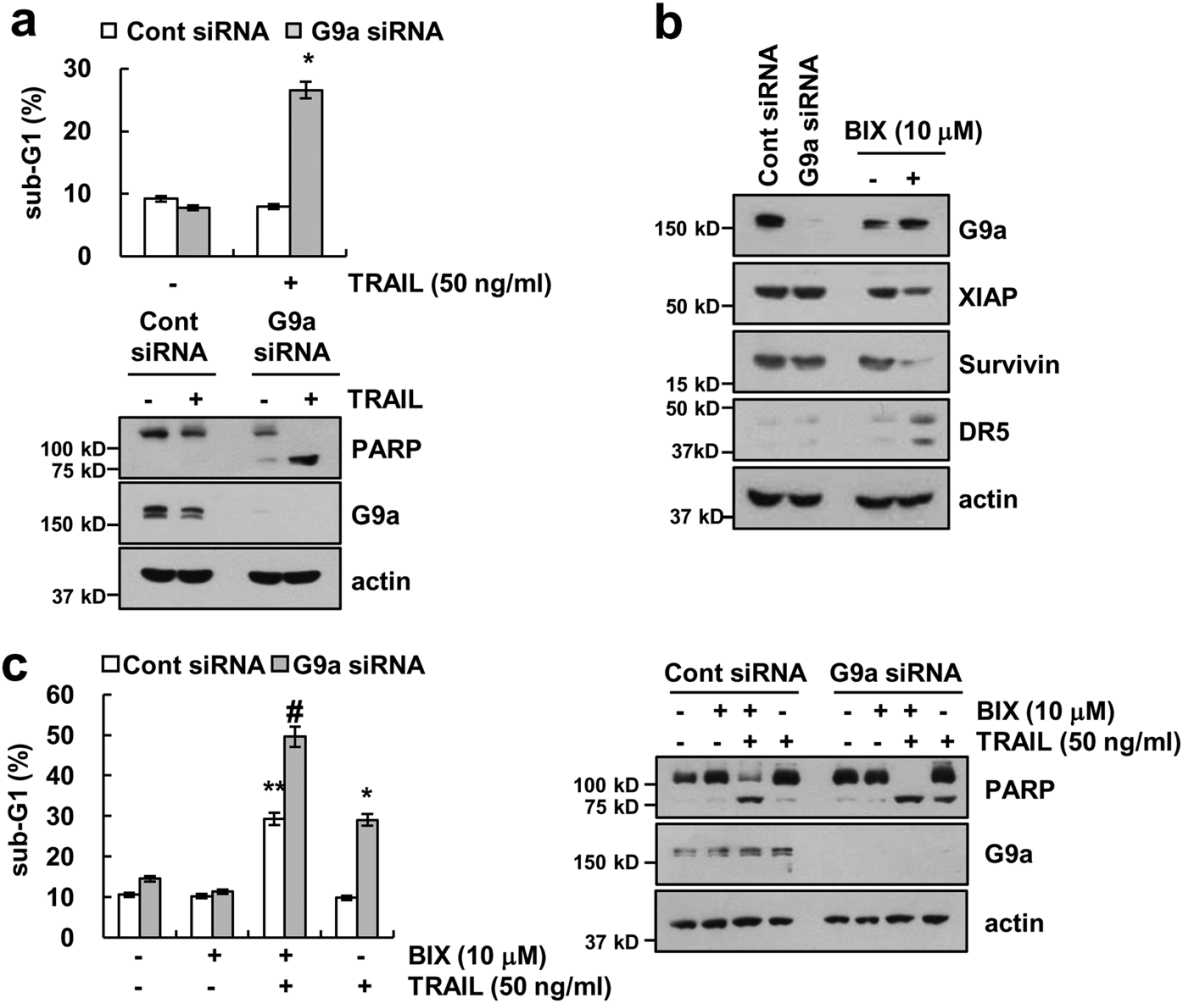
Knockdown of G9a sensitizes Caki cells to TRAIL-mediated apoptosis. **a** Caki cells were transiently transfected control siRNA (Cont siRNA) or G9a siRNA. Twenty-four hours after transfection, cells were treated with 50 ng/ml TRAIL for 24 h. Apoptosis was analyzed as a sub-G1 population by flow cytometry (upper panel). The protein levels of PARP, G9a, and actin were determined by western blotting. (lower panel). **b** Caki cells were transiently transfected control siRNA (Cont siRNA) or G9a siRNA or treated with 10 μM BIX for 24 h. The protein levels of G9a, XIAP, survivin, DR5, and actin were determined by western blotting. **c** Caki cells were transiently transfected control siRNA (Cont siRNA) or G9a siRNA. Twenty-four hours after transfection, cells were treated 50 ng/ml TRAIL in the presence or absence of 10 μM BIX for 24 h. Apoptosis was analyzed as a sub-G1 population by flow cytometry. The protein levels of PARP, G9a, and actin were determined by western blotting. The level of actin was used as a loading control. The values in a represent the mean ± SD from three independent samples; *p < 0.01 compared with TRAIL-treated control siRNA. **p < 0.01 compared with control-control siRNA.^ #^*p < *0.01 compared with BIX plus TRAIL-treated control siRNA

To investigate whether the apoptosis induced by combined treatment with BIX plus TRAIL is dependent on the inhibition of histone methyltransferase G9a, Caki cells were transfected with G9a siRNA. As shown in Fig. 6a, downregulation of G9a by siRNA markedly enhanced TRAIL-mediated apoptosis and PARP cleavage. We next examined whether the downregulation of G9a regulated XIAP, survivin, and DR5, similar to observed with the effect of BIX treatment. However, downregulation of G9a by siRNA did not induce downregulation of XIAP and survivin expression, as well as upregulation of DR5 expression (Fig. 6b). To further confirm these findings, we investigated the effect of BIX on TRAIL-mediated apoptosis in G9a-downregulated cells by siRNA. As shown in Fig. 6c, combined treatment with BIX plus TRAIL more induced apoptosis in G9a knockdown cells, compared with control cells. Therefore, these data indicate that inhibition of G9a by gene silencing (siRNA) or a pharmacological inhibitor (BIX) could enhance TRAIL-mediated apoptosis via a different molecular mechanism.

### Effect of combined treatment with BIX and TRAIL on apoptosis in other cancer cells and normal cells

**Fig. 7:**
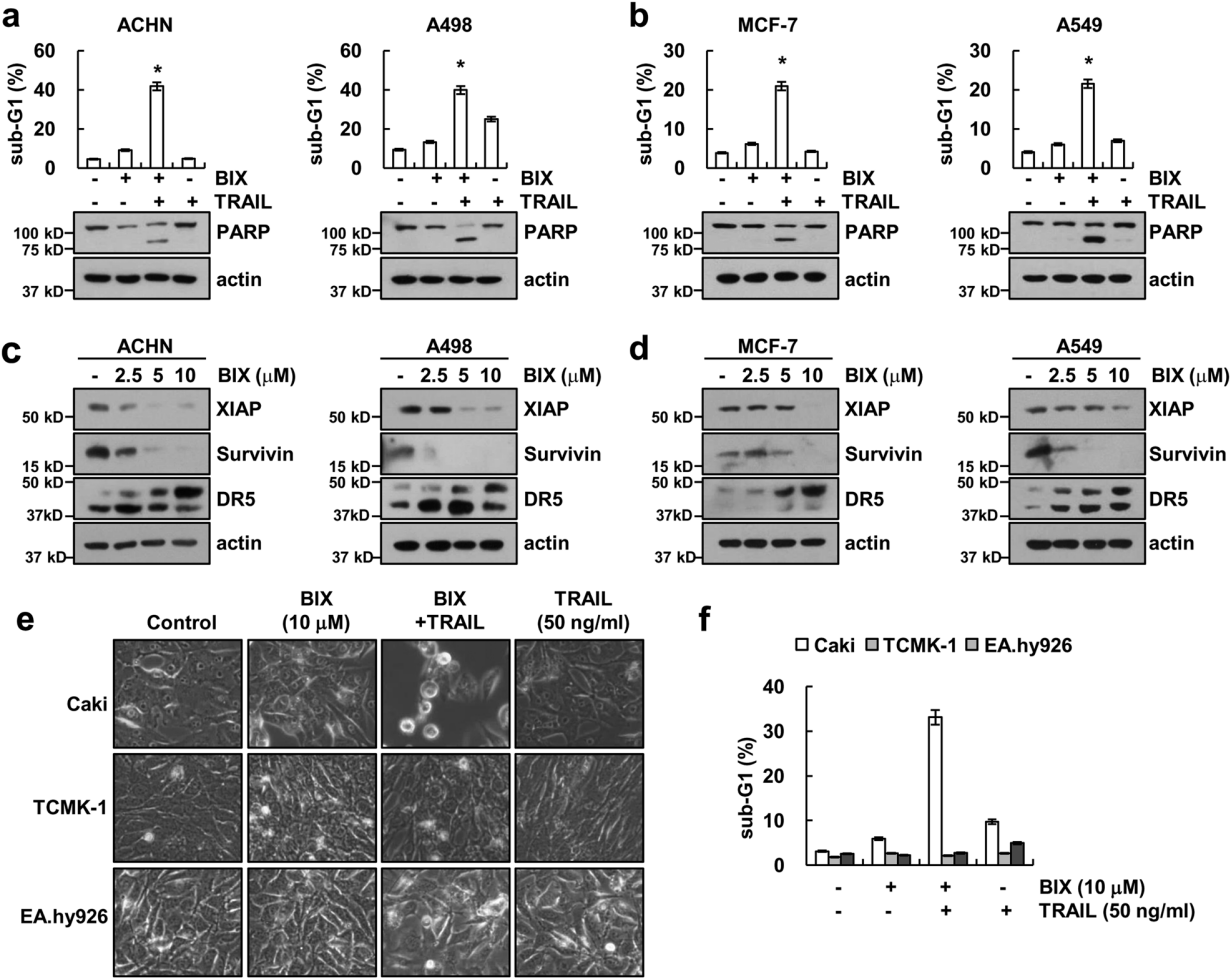
Effect of the combined treatment with BIX-01294 and TRAIL on apoptosis in other cancer and normal cells. **a,** **b** Renal carcinoma (ACHN and A498), breast carcinoma (MCF-7) and lung carcinoma (A549) were treated with 50 ng/ml TRAIL in the presence or absence of 10 μM BIX for 24 h. Apoptosis was analyzed as a sub-G1 population by flow cytometry (upper panel). The protein levels of PARP and actin were determined by western blotting. The level of actin was used as a loading control (lower panel). **c,** **d** Renal carcinoma (ACHN and A498), breast carcinoma (MCF-7), and lung carcinoma (A549) were treated with the indicated concentration of BIX for 24 h. The protein levels of XIAP, survivin, DR5, and actin were determined by western blotting. The level of actin was used as a loading control. **e,** **f** Caki, TCMK-1, and EA.hy926 cells were treated with 50 ng/ml TRAIL in the presence or absence of 10 μM BIX for 24 h. The cell morphology was examined using interference light microscopy **e**. The level of apoptosis was assessed by measuring the sub-G1 fraction using flow cytometry **f**. The values in **a**, **b**, and **f** represent the mean ± SD from three independent samples; *p < 0.01 compared with the control

We further investigated whether combined treatment with BIX and TRAIL-induced apoptosis in other cancer cells and normal cells. As shown in Figs. 7a, b, we found that combined treatment with BIX and TRAIL increased apoptosis and PARP cleavage in renal carcinoma cells (ACHN and A498), breast carcinoma cells (MCF-7), and human lung carcinoma cells (A549). Furthermore, BIX induced downregulation of XIAP and survivin and upregulation of DR5 expression (Figs. 7c, d). However, combined treatment with BIX plus TRAIL had no effect on apoptosis and morphological changes in normal mouse kidney cells (TCMK-1) and normal human umbilical vein (EA.hy926) cells (Figs. 7e, f), although human TRAIL is known to bind to murine TRAIL receptor^28^. Taken together, these data suggest that BIX could selectively sensitize TRAIL-mediated apoptosis in cancer cells.

## Discussion

In this study, we found that BIX enhances TRAIL-induced apoptosis in various cancer cell lines, but not in normal cells. BIX induced downregulation of survivin at the post-translational level and upregulation of DR5 expression at the transcriptional level. Ectopic expression of survivin or downregulation of DR5 by siRNA inhibited BIX plus TRAIL-induced apoptosis. Therefore, these data suggest that BIX may be used as a powerful sensitizer of TRAIL.

Previous studies have reported that 5 μM BIX inhibited the proliferation and induced apoptosis through downregulation of Bcl-2 expression and upregulation of Bax expression in U251 glioma cells^29^. However, twofold higher concentrations of BIX treatment did not modulate Bcl-2 expression in Caki cells (Fig. 1g). Interestingly, we observed that BIX decreased survivin protein expression at the post-translational level (Fig. 3b). Survivin, a member of the family of IAPs, is a short-lived protein that prevents apoptosis by inhibiting the activation of caspase. Survivin was regulated at the transcriptional and post-translational levels. The ubiquitin–proteasome pathway is involved in post-translational regulation of survivin via cell cycle-dependent manner^30^ and depletion of K-Ras^31^. Arora et al. reported that increase of XIAP, an E3 ubiquitin ligase, is associated with proteasome-dependent survivin downregulation^32^. BIX markedly induced downregulation of XIAP protein expression in Caki cells (Fig. 1a). Therefore, we need further study to identify the E3 ubiquitin ligase involved in BIX-mediated survivin protein degradation.

BIX induced upregulation of DR5 expression at the transcriptional level, and BIX-induced DR5 upregulation plays a role on TRAIL-mediated apoptosis (Figs. 4d, e). BIX induced Beclin-1 expression through nuclear factor κB (NF-κB)-dependent transcriptional activation in breast cancer^16^. It is well known that DR5 expression is mainly regulated by the Sp1, CHOP, p53, and NF-κB transcription factors^33–36^. Chen et al. demonstrated that the NF-κB element on the DR5 first intron region plays a critical role in proteasome inhibitor-induced DR5 expression^36^. We observed that BIX increased NF-κB transcriptional activity, but NF-κB inhibitors (Bay and PDTC) did not block BIX-induced upregulation of DR5 expression (data not shown). Therefore, these data suggest that BIX-induced DR5 expression is not associated with NF-κB activation.Please define NF-κB at its first mention in text.

Previous studies reported that BIX generates ROS production in breast cancer cells^15, 16^. BIX markedly accumulated intracellular ROS, and pretreatment with NAC inhibited BIX-mediated cell death^15^. BIX also generated intracellular ROS in human renal carcinoma Caki cells (Fig. 5a), but ROS scavengers were not affected by combined treatment with BIX and TRAIL and BIX-mediated modulation of XIAP, survivin, and DR5 expression (Figs. 5b, c). Thus, these data indicate that BIX-mediated TRAIL sensitization is independent of ROS signaling pathway.

BIX has been known as a G9a inhibitor, but we found that BIX also sensitized TRAIL-mediated apoptosis in G9a knockdown cells (Fig. 6c). Furthermore, BIX induced downregulation of survivin and upregulation of DR5 expression, but knockdown of G9a expression did not change expression of survivin and DR5 (Fig. 6b). Therefore, it is possible that BIX enhances sensitivity to TRAIL as a consequence of its off-target effect.

In summary, these findings supported that BIX sensitizes TRAIL-induced apoptotic cell death through downregulation of survivin at the post-translational level and upregulation of DR5 at the transcriptional level in Caki cell, but not in normal cells. Therefore, we suggest that BIX could be a therapeutic strategy to overcome TRAIL resistance in cancer cells.

## Materials and methods

### Cell cultures and materials

Human renal carcinoma (Caki, ACHN, and A498), human breast carcinoma cells (MCF-7), human lung carcinoma cells (A549), normal human umbilical vein cell (EA.hy926), and normal mouse kidney cells (TCMK-1) were obtained from the American Type Culture Collection (Manassas, VA, USA). All cells were cultured in Dulbecco’s modified Eagle’s medium containing 10% fetal bovine serum, 20 mM HEPES buffer, 100 U/ml penicillin, 100 μg/ml streptomycin, and 100 μg/ml gentamicin. The PCR primers were purchased from Macrogen (Seoul, Korea). Recombinant human TRAIL and BIX were purchased from Sigma Chemical Co. (St. Louis, MO, USA). z-VAD-fmk and anti-survivin antibodies were purchased from R&D system (Minneapolis, MN, USA). Anti-Mcl-1 and anti-cIAP2 antibodies were purchased from Santa Cruz Biotechnology (Dallas, TX, USA). Anti-Bim and anti-XIAP antibodies were purchased from BD Biosciences (San Jose, CA, USA). Anti-PARP, anti-Bcl-2, anti-Bcl-xL, anti-DR5, and anti-G9a antibodies were purchased from Cell Signaling Technology (Beverly, MA, USA). Anti-actin antibody and other chemicals were purchased from Sigma Chemical Co.

### Flow cytometry analysis

For flow cytometry, the cells were resuspended in 100 μl of phosphate-buffered saline (PBS), and 200 μl of 95% ethanol was added while the cells were being vortexed. Then, the cells were incubated at 4 °C for 1 h, washed with PBS, resuspended in 250 μl of 1.12% sodium citrate buffer (pH 8.4) with 12.5 μg of RNase, and incubated for an additional 30 min at 37 °C. The cellular DNA was then stained by adding 200 μl of a propidium iodide solution (50 μg/ml) to the cells for 30 min at room temperature. The stained cells were analyzed by fluorescent-activated cell sorting on a FACScan flow cytometer (BD Biosciences) to determine the relative DNA content, which was based on the red fluorescence intensity.

### Western blot analysis

Cells were washed with cold PBS and lysed on ice in 50 μL of lysis buffer (50 mM Tris-HCl, 1 mM EGTA, 1% Triton X-100, 1 mM phenylmethylsulfonyl fluoride, pH 7.5). Lysates were centrifuged at 10,000 × *g* for 15 min at 4 °C, and the supernatant fractions were collected. Proteins were separated by sodium dodecyl sulfate–polyacrylamide gel electrophoresis and transferred to an Immobilon-P membrane (GE Healthcare Life Science, Pittsburgh, PO, USA). Specific proteins were detected using an enhanced chemiluminescence Western blot kit (EMD Millipore, Darmstadt, Germany), according to the manufacturer’s instructions.

### 4′,6′-Diamidino-2-phenylindole (DAPI) staining for nuclei condensation and fragmentation

To examine cellular nuclei, the cells were fixed with 1% paraformaldehyde on glass slides for 30 min at room temperature. After the fixation, the cells were washed with PBS, and a 300 nM DAPI solution (Roche, Basel, Switzerland) was added to the fixed cells for 5 min. After the nuclei were stained, the cells were examined by fluorescence microscopy (Carl Zeiss, Jena, Germany).

### Cell death assessment by DNA fragmentation assay

The cell death detection ELISA plus kit (Boehringer Mannheim, Indianapolis, IN, USA) was used for assessing apoptotic activity by detecting fragmented DNA within the nucleus in BIX-treated, TRAIL-treated, and combination with BIX and TRAIL-treated cells. Briefly, each culture plate was centrifuged for 10 min at 200 × *g*, the supernatant was removed, and the pellet was lysed for 30 min using lysis buffer that was included in the kit. After centrifuging the plate again at 200 × *g* for 10 min, the supernatant (cytoplasmic fraction) that contained the cytoplasmic histone-associated DNA fragments was collected and incubated with an immobilized anti-histone antibody. The reaction products were incubated with a peroxidase substrate for 5 min and measured by spectrophotometry at 405 and 490 nm (reference wavelength) with a microplate reader (BMG Labtech, Ortenberg, Germany). The signals in the wells containing the substrate alone were subtracted as the background.

### Asp-Glu-Val-Asp-ase (DEVDase) activity assay

To evaluate DEVDase activity, cell lysates were prepared after their respective treatments with TRAIL in the presence or absence of BIX. Assays were performed in 96-well microtiter plates by incubating 20 μg of cell lysates in 100 μl of reaction buffer (1% NP-40, 20 mM Tris-HCl, pH 7.5, 137 mM NaCl, 10% glycerol) containing a caspase substrate (Asp-Glu-Val-Asp-chromophore-p-nitroanilide (DEVD-pNA), EMD Millipore) at 5 μM. Lysates were incubated at 37 °C for 2 h. Thereafter, the absorbance at 405 nm was measured with a spectrophotometer (BMG Labtech).

### Reverse transcription polymerase chain reaction (RT-PCR) and quantitative PCR (qPCR)

Total RNA was isolated using the TriZol reagent (Life Technologies, Gaithersburg, MD, USA), and the complementary DNA (cDNA) was prepared using M-MLV reverse transcriptase (Gibco-BRL, Gaithersburg, MD, USA) according to the manufacturer’s instructions^37, 38^. The following primers were used for the amplification of human XIAP, survivin, DR5 and actin: XIAP (forward) 5′-AGCATCAACACTGGCACGAGCA-3′ and (reverse) 5′-GTGTCGCCTGTGTTCTGACCAG-3′; survivin (forward) 5′-GGACCACCGCATCTCTACAT-3′ and (reverse) 5′-GCACTTTCTTCGCAGTTTCC-3′; DR5 (forward) 5′-AAGACCCTTGTGCTCGTTGT-3′ and (reverse) 5′-GACACATTCGATGTCACTCCA-3′; and actin (forward) 5′-GGCATCGTCACCAACTGGGAC-3′ and (reverse) 5′-CGATTTCCCGCTCGGCCGTGG-3′. The PCR amplification was carried out using the following cycling conditions: 94 °C for 3 min followed by 17 (actin) or 28 cycles (XIAP, survivin and DR5) of 94 °C for 40 s, 56 °C for 40 s, 72 °C for 1 min, and a final extension at 72 °C for 5 min. The amplified products were separated by electrophoresis on a 1.5% agarose gel and detected under ultraviolet light. For qPCR, cDNA and forward/reverse primers (200 nM) were added to 2 × KAPA SYBR Fast master mix, and reactions were performed on LightCycler 480 real-time amplification instrument (Roche). The following primers were used for the amplification of human XIAP, survivin, DR5 and actin: XIAP (forward) 5′-AGCATCAACACTGGCACGAGCA-3′ and (reverse) 5′-GTGTCGCCTGTGTTCTGACCAG-3′; survivin (forward) 5′-TTCTCAAGGACCACCGCATC-3′ and (reverse) 5′-GTTTCCTTTGCATGGGGTCG-3′; DR5 (forward) 5′-AGACCCTTGTGCTCGTTGTC-3′ and (reverse) 5′-TTGTTGGGTGATCAGAGCAG-3′; and actin (forward) 5′-CTACAATGAGCTGCGTGTG-3′ and (reverse) 5′-TGGGGTGTTGAAGGTCTC-3′. Threshold cycle number (Ct) of each gene was calculated, and actin was used as reference genes. Delta-delta Ct values of genes were presented as relative fold induction.

### Proteasome activity assay

The chymotryptic proteasome activities were measured with Suc-LLVY-AMC (chymotryptic substrate, Biomol International, Plymouth Meeting, PA, USA). The cells were collected, washed with PBS, and lysed. A mixture containing 1 μg protein of the cell lysate in 100 mM Tris-HCl (pH 8.0), 10 mM MgCl_2_, and 2 mM ATP was incubated at 37 °C for 30 min with 50 μM Suc-LLVY-AMC. The enzyme activity was measured with a fluorometric plate reader at an excitation wavelength of 380 nm and an emission wavelength of 440 nm.

### Stable transfection in caki cells

Caki cells were transfected in a stable manner with the pcDNA3.1/survivin-3xflag or control plasmid pcDNA 3.1 vector using Lipofectamine^TM^ 2000, as prescribed by the manufacturer (Invitrogen, Carlsbad, CA, USA). After 48 h of incubation, transfected cells were selected in primary cell culture medium containing 700 μg/mL G418 (Invitrogen). After 2 or 3 weeks, single independent clones were randomly isolated, and each individual clone was plated separately. After clonal expansion, cells from each independent clone were tested for survivin expression by immunoblotting.

### Analysis of cell surface DR5

Cells were detached with 0.5 mM EDTA and washed three times with PBS. Washed cells were suspended in 200 μl of PBS, and primary antibody was added and incubated for 1 h at room temperature. Then, the cells were washed twice with PBS, resuspended in 200 μl of PBS, and incubated with fluorescein isothiocyanate-conjugated secondary antibody for 30 min at room temperature. Unbound secondary antibody was removed by centrifugation, and cells were resuspended in 500 μl of PBS. Cell surface expression of DR5 was determined by flow cytometry.

### Small interfering RNA

The G9a siRNA duplexes used in this study were purchased from Santa Cruz Biotechnology. The GFP (control) siRNA and DR5 siRNA duplexes were purchased from Bioneer (Daejeon, Korea). Cells were transfected with siRNA oligonucleotides using Lipofectamine® RNAiMAX Reagent (Invitrogen), according to the manufacturer’s recommendations.

### Statistical analysis

The data were analyzed using a one-way analysis of variance and post hoc comparisons (Student–Newman–Keuls), using the Statistical Package for Social Sciences 22.0 software (SPSS Inc., Chicago, IL, USA).
